# A Comparative Study of Narrow/Ultra-Wideband Microwave Sensors for the Continuous Monitoring of Vital Signs and Lung Water Level

**DOI:** 10.3390/s24051658

**Published:** 2024-03-04

**Authors:** Anwer S. Abd El-Hameed, Dalia M. Elsheakh, Gomaa M. Elashry, Esmat A. Abdallah

**Affiliations:** 1 Microstrip Department, Electronics Research Institute (ERI), El Nozha 11843, Egypt; daliaelsheakh@eri.sci.eg (D.M.E.); gomaa.m.ashry@eri.sci.eg (G.M.E.); esmataa2@hotmail.com (E.A.A.); 2 Computer and Communication Department, Faculty of Engineering, Nahda University, Beni Suef 62746, Egypt; 3 Electrical Department, Faculty of Engineering and Technology, Badr University in Cairo, Badr 11829, Egypt

**Keywords:** microwave antenna sensor (MAS), vital sign detection (VSD), NB, ultra-wideband (UWB) sensor, flexible, textile, lung water level (LWL), specific absorption rate (SAR)

## Abstract

This article presents an in-depth investigation of wearable microwave antenna sensors (MASs) used for vital sign detection (VSD) and lung water level (LWL) monitoring. The study looked at two different types of MASs, narrowband (NB) and ultra-wideband (UWB), to decide which one was better. Unlike recent wearable respiratory sensors, these antennas are simple in design, low-profile, and affordable. The narrowband sensor employs an offset-feed microstrip transmission line, which has a bandwidth of 240 MHz at −10 dB reflection coefficient for the textile substrate. The UWB microwave sensor uses a CPW-fed line to excite an unbalanced U-shaped radiator, offering an extended simulated operating bandwidth from 1.5 to 10 GHz with impedance matching ≤−10 dB. Both types of microwave sensors are designed on a flexible RO 3003 substrate and textile conductive fabric attached to a cotton substrate. The specific absorption rate (SAR) of the sensors is measured at different resonant frequencies on 1 g and 10 g of tissue, according to the IEEE C95.3 standard, and both sensors meet the standard limit of 1.6 W/kg and 2 W/kg, respectively. A simple peak-detection algorithm is used to demonstrate high accuracy in the detection of respiration, heartbeat, and lung water content. Based on the experimental results on a child and an adult volunteer, it can be concluded that UWB MASs offer superior performance when compared to NB sensors.

## 1. Introduction

The growing number of old people in the population has led to a surge in research into remote healthcare technologies. One of the most promising enabling technologies is wireless vital sign monitoring, in which sensors attached to the body can transmit information about a person’s heart or breathing rate. These data can provide valuable insights into a person’s health status [1].

The use of wearable technologies to continuously monitor patients has revolutionized healthcare outside the hospital, according to [2]. Researchers have proposed a number of approaches to tracking breathing patterns [3]. One possible technique for monitoring this vital physiological parameter in real time is the use of sensors. However, the effectiveness of different methods may vary depending on factors such as the type of sensor and breathing parameters, the location of the sensor, the data processing techniques, the analysis software used, and performance metrics.

Since it can be used to accurately identify pulmonary edema at an early stage and as a follow-up for treatment in critically burned and heart surgery patients, the lung water level is an essential medical metric [4]. Although the measures of the lung water level offer a wealth of therapeutic knowledge, a thorough examination of the literature demonstrates that there are few accurate, noninvasive, affordable, and simple-to-use medical sensors to measure the variations in lung water level [5].

Most wearable devices available today are either smart watches or fitness bands that can provide users with information about their physical activity, movements, and some vital signs [6]. Despite their popularity, these devices have limited accuracy, validity, and reliability in real clinical settings. Additionally, their bulky and non-flexible designs make them uncomfortable to wear for extended periods of time, and their high-power consumption limits their battery life and accuracy [7]. Other limitations include the restricted number of anatomical locations for sensor placement, motion artifacts, and difficulties in interpreting the large amount of generated data. To fully realize the potential of digital and personalized healthcare, these challenges need to be addressed through improved technology and market adaptation [8].

Methods of measuring respiratory parameters involve using sensors that must be in contact with the patient’s body [9]. These methods analyze several parameters sampled from the flow of air during inhalation and/or exhalation. Alternatively, methods based on measuring chest and abdominal movements have been used [10]. These sensors can be directly attached to the torso or integrated into clothing fibers, and can be resistive, capacitive, or inductive [11]. According to several studies [7,8,9], regularly monitoring basic vital signs such as heart rate and respiration rate is highly beneficial in detecting various medical conditions. While there are numerous solutions available today that can provide continuous heart rate monitoring, only a limited number can also offer information on respiration rate; an example includes a circularly polarized (CP) compact wearable dielectric resonator antenna (DRA) [12]. This information is crucial in preventing respiratory disorders such as asthma, pneumonia, chronic obstructive pulmonary disease (COPD), and sleep apnea, as it improves diagnostic capabilities.

The Cardio-Pulmonary Stethoscope (CPS) [13] technology, a unique “Chest Patch” sensor device for non-invasive and continuous monitoring of vital signs and LWL, has been created and patented by the team. The device complies with FCC safety standards and makes use of inexpensive 915 MHz RF transceivers [14].

There are different types of sensors used as coupled and radiating MASs; however, radiator sensors are not desirable due to radiation and the effect of radiation on the human body. However, the proposed antenna sensors are designed with low SAR value, so these sensors have less effect on the human body. Ultra-wideband (UWB) and NB antennas are increasingly being used for vital sign detection (VSD) due to their ability to operate at low power and their ability to provide accurate and reliable measurements. UWB antennas are capable of transmitting and receiving short pulses of electromagnetic waves that can penetrate through obstacles and reflect off the human body to provide highly precise measurements of vital signs such as heart rate and respiration rate [2,15,16,17,18,19,20,21,22,23,24].

Narrow band antennas, on the other hand, operate at a fixed frequency, allowing them to provide highly accurate measurements with a low error rate. Both types of antennas have been used in various applications, including wearable devices and healthcare systems [25,26,27,28,29,30]. Several studies have demonstrated the effectiveness of UWB and NB antennas for vital sign detection [31,32,33,34], paving the way for their widespread use in healthcare and wellness monitoring applications.

Studying the difference between narrow and UWB band MAS for VSD and LWL is crucial in developing accurate and reliable wearable systems. Both types of antennas have advantages and disadvantages in terms of their bandwidth, signal-to-noise ratio, and interference levels. Understanding these differences can help in selecting the most appropriate antenna for a particular application, which can impact the accuracy and reliability of the vital sign measurements. Furthermore, the choice of antenna can impact the overall design of the wearable system, including size, power consumption, and cost. Therefore, research on the comparative performance of narrow and UWB band MAS could aid in the development of effective and efficient vital sign and LWL detection systems.

Accordingly, this paper aims to conduct a comprehensive analysis of the efficacy of narrowband and UWB antennas in VSD and LWL. This study will involve the use of both conventional flexible dielectric substrate and wearable cotton substrate in designing the antennas, and their performance will be evaluated. Moreover, a complete system for vital sign detection will be proposed and employed in assessing the antenna performance. The safety of both types of antennas will also be evaluated by incorporating SAR measurements.

The rest of the paper is organized as follows: the design of the microwave sensors is presented in Section 2 for both NB and UWB antennas. The effect of the human body on the antenna performance is described in Section 3. Section 4 presents the results of the SAR measurements while Section 5 describes the proposed system for vital sign detection. Section 6 presents the application of the NB and UWB sensors in measuring the lung water level (LWL) and compares their performance. Section 7 introduces the discussion and comparison with previously published research, while the conclusion is given in Section 8.

## 2. Design of Microwave Sensors

The design of microwave sensors involves the careful consideration of the sensing mechanism, the choice of materials and components, and the overall system architecture. Antenna design is critical to ensure the high sensitivity and selectivity of the sensor. In this section, we will present the design details and performance of both proposed NB and UWB antennas.

### 2.1. Narrowband Antenna (NB)

An effective method of eliminating harmonic frequencies is to use narrowband antennas, which employ harmonic suppression and size reduction techniques. Conventional antennas are unsuitable for modern wireless and portable systems, which has made narrowband antennas popular in communication systems. The microstrip patch antenna has become a favorite among antenna designers for commercial mobile and wireless communication systems due to its many advantages.

**Figure 1:**
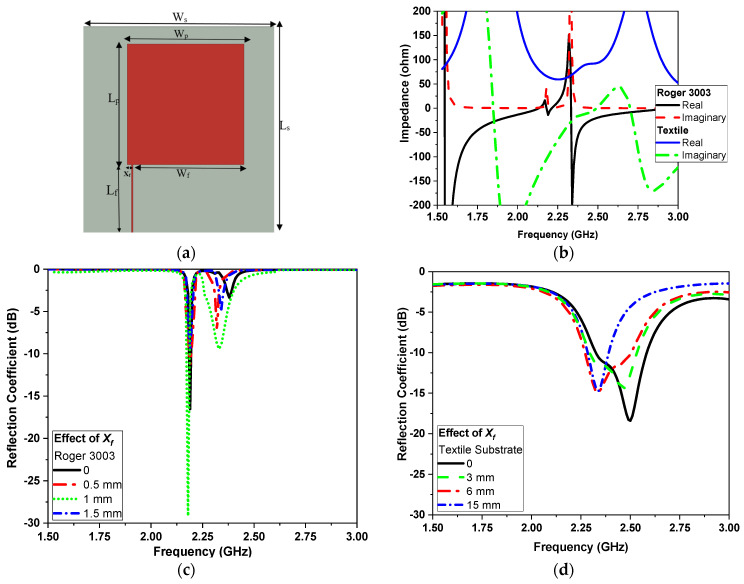
(**a**) Configuration of the offset feed transmission line microstrip sensor, (**b**) input impedance (real and imaginary) for both the flexible and the textile substrate and the effect of the location X_f_ on (**c**) the flexible and (**d**) the textile substrate.

As depicted in Figure 1, a microstrip patch is connected directly to a microstrip transmission line. Usually, the impedance at the patch edge is much higher than 50 Ohms, resulting in an impedance mismatch. To solve this problem, quarter-wavelength transformers can be used to transform the high input impedance into a 50-ohm line. However, this technique is only effective for a very narrow bandwidth. Therefore, to achieve a reasonable bandwidth, we used the offset feeding technique. The dimensions of the rectangular microstrip antenna are critical to its performance. Efficient radiation from the antenna requires specific length and width dimensions, which can be determined using Equations (1)–(3); The width, W, is [35]

(1) $$W=\frac{C}{2f}\times \sqrt{\frac{2}{\varepsilon r+1}}$$

The resonant frequency (f) and dielectric constant of the substrate (εr) are crucial parameters in the design of the antenna; C is the velocity of light in free-space. The length, L, is

(2) $$L=\frac{c}{2f}{\left(\frac{\varepsilon_{r}+1}{2}+\frac{\varepsilon_{r}-1}{2}\sqrt{1+12\frac{h}{W}}\right)}^{-\frac{1}{2}}-2∆L$$

(3) $$∆L=0.412h\frac{{(\varepsilon }_{reff}+0.3)(\frac{W}{h}+0.264)}{{(\varepsilon }_{reff}-0.258)(\frac{W}{h}+0.8)}$$

where h is the substrate thickness, and $\varepsilon_{reff}$ is the effective dielectric constant.

The design process begins by selecting a flexible substrate and then transferring the design to a textile for it to be friendly with the human body and easy for wearing. To achieve this, a flexible substrate of Rogers 3003 with a thickness of 0.17 mm and a dielectric constant of 3.5 and tan δ = 0.0002 is chosen. The 3D electromagnetic field simulator Computer Simulation Technology (CST) Studio Suite ver. 2021 is used [36]. The antenna with an offset feeding line is utilized to obtain a reasonable bandwidth, and the dimensions of the traditional microstrip antenna are calculated using the equation given in references [35,37]. The input impedance of the offset feed microstrip antenna is matched at 2.25 GHz and 2.4 GHz. Figure 1b illustrates a graphical representation of the real and imaginary parts of the input impedance performance for both substrate technologies of the antenna. The optimized dimensions for the sensor are outlined in Table 1, considering both flexible substrate and textile technologies. The transmission line feed has a length and width of 12 mm and 0.3 mm for the flexible substrate and 20 mm and 2 mm for the textile. The impact of the 50 Ω transmission line feeding X_f_ location is depicted in Figure 1c for the flexible substrate and Figure 1d for the textile.

**Figure 2:**
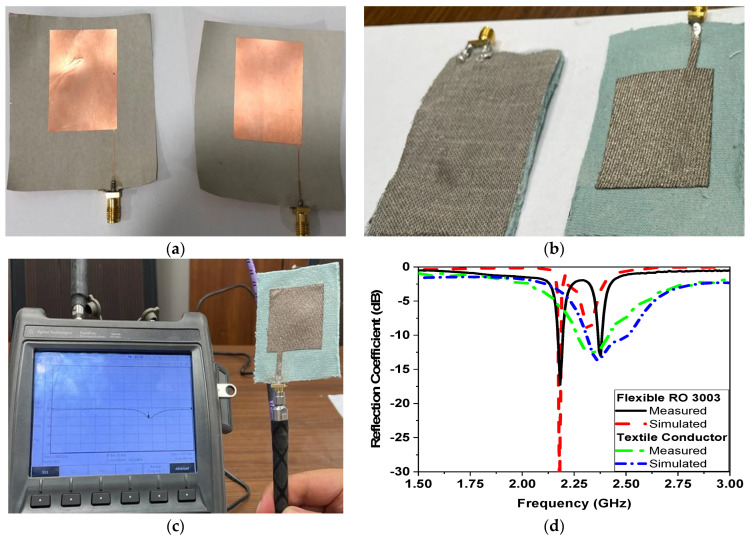
Photo of fabricated antenna. (**a**) Flexible substrate, (**b**) conductive fabric, (**c**) measurement setup, and (**d**) simulated and measured |S11|.

The narrowband antenna design suggested in this study is implemented on both flexible and textile substrates, as demonstrated in Figure 2a,b. The measured bandwidth at the impedance matching ≤−10 dB is 100 KHz and 240 MHz for the flexible and the textile substrate, respectively. The performance of the antenna is assessed using a portable vector network analyzer made by Agilent Field Fox N9918A 26.5 GHz, as shown in Figure 2c. The fabrication process involves photolithography techniques carried out in the Microstrip Department, Electronics Research Institute, Cairo, Egypt, while the textile sensor is fabricated by using a high-quality laser cutting machine. Figure 2d displays a comparison between the |S11| magnitude obtained from the simulations and the measured results. It is evident that the antenna functions at the central frequency of 2.45 GHz, which is specifically assigned for ISM (industrial, scientific, and medical) applications.

### 2.2. Ultra-Wideband (UWB) Microwave Sensor

This section introduces an UWB sensor for detecting vital human signs, which is designed to be conformal and consists of a wideband monopole antenna with a CPW feed line. The proposed design features an asymmetric U-shaped radiating element with different arm lengths and a modified ground plane.

**Figure 3:**
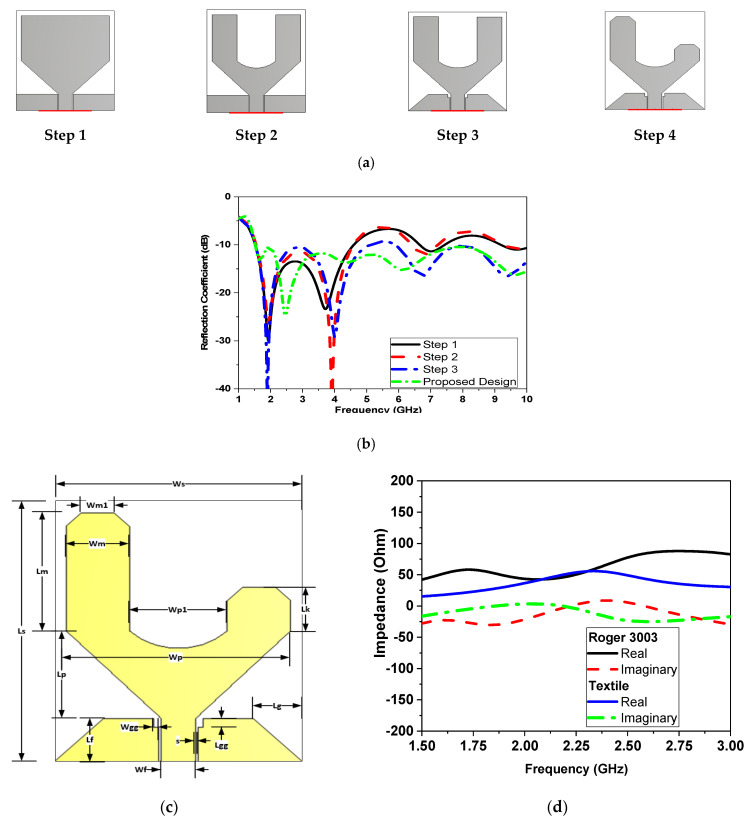
(**a**) Design steps, (**b**) simulated |S11| of design steps, (**c**) configuration of the UWB monopole antenna, and (**d**) impedance (real and imaginary).

The initial design of the UWB antenna took the form of a trapezoidal monopole, as illustrated in Figure 3a during step 1. The bandwidth covered the range from 1.5 GHz to 4.4 GHz with |S11| ≤ −10 dB. Subsequently, a U-shaped slot was introduced in step 2 to enhance matching, bringing it from −25 dB to −40 dB at the resonant frequency of 4 GHz. In step 3, ground chamfering was applied to further refine matching and extend the frequency band. Step 4 involved the addition of a shortened arm, creating a U-shaped configuration with unequal arms, and chamfering both arms for improved performance. Figure 3 displays the reflection coefficient responses for all these preceding steps.

This design enables the operating bandwidth to be extended from 1.5 GHz to more than 10 GHz while maintaining a reflection coefficient |S11| of less than or equal to −10 dB. The antenna is fabricated using a flexible substrate of Rogers 3003 with a thickness of 0.17 mm and a dielectric constant of 3 and tan δ = 0.0002. The same design is implemented by using the same optimized dimensions as shown in Table 2 on a biodegradable conductive textile attached to a cotton substrate with dimensions of 60 × 60 mm^2^. The radiator and ground plane dimensions are presented in Figure 3c and Table 2, with a 50 Ω transmission line feed length and width for both substrate technologies used of 12 mm and 0.3 mm, and 20 mm and 2 mm, respectively.

In the same electromagnetic field simulators, CST is used to complete these simulations. The antenna input impedance, real and imaginary parts for both technologies of flexible and textile substrates are shown in Figure 3a,b, respectively. The antenna operates in the 2.45 GHz ISM band. Comprehensive simulations are carried out on all the parameters of the antenna to study their individual effects.

**Figure 4:**
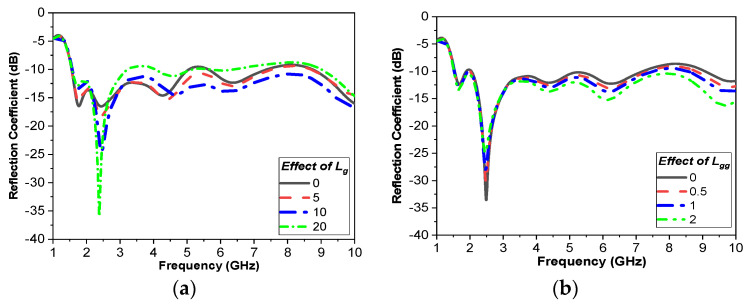

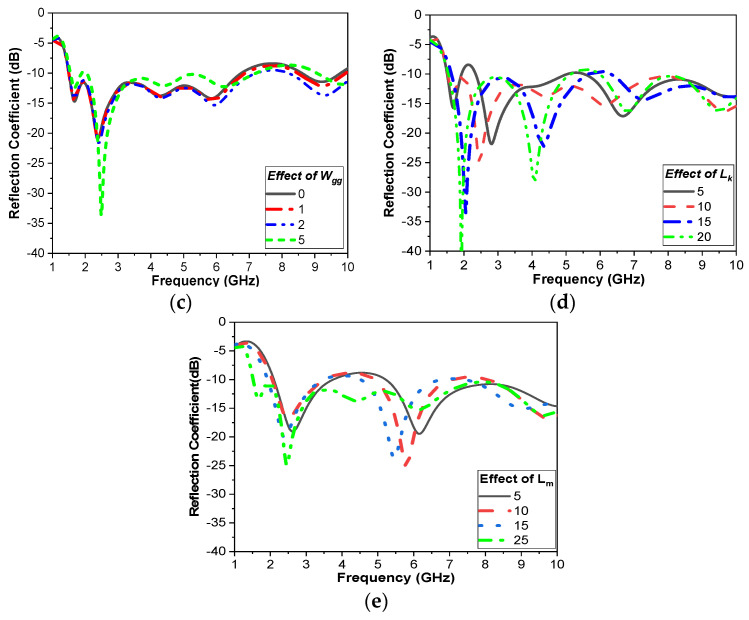
|S11| versus frequency for different parametric sweeps. (**a**) Effect of L_g_. (**b**) Effect of L_gg_. (**c**) Effect of W_gg_.(**d**) Effect of L_k_. (**e**) Effect of L_m_.

**Figure 5:**
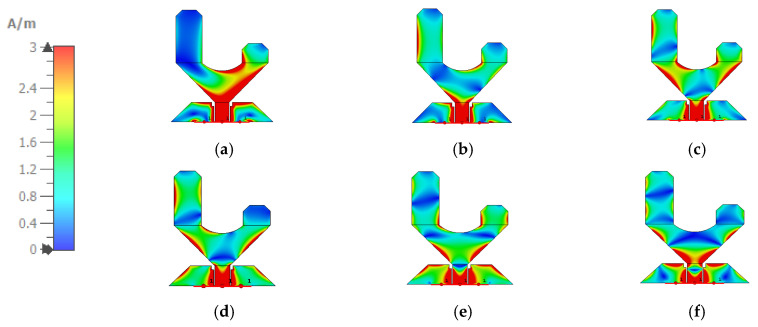
Surface current density distribution of the proposed monopole at different frequencies: (**a**) 2.4 GHz, (**b**) 3.5 GHz, (**c**) 5.2 GHz, (**d**) 6 GHz, (**e**) 7.5 GHz, and (**f**) 10 GHz.

The influence of *L_g_*, *L_gg_*, *W_gg_*, *L_k_*, and *L_m_* on the performance of the antenna is depicted in Figure 4a–e. Figure 4a shows the effect of *L_g_*; by increasing this length, the microwave sensor matching improves. When *L_gg_* and *W_gg_* are increased, the response is slightly changed, especially at frequencies higher than 4 GHz, as shown in Figure 4b,c. Also, when *L_k_* is increased, the bandwidth of the microwave sensor improves, as shown in Figure 4d. Moreover, Figure 4e shows that as *L_m_* is increased, the fundamental frequency is reduced. Furthermore, to demonstrate the contribution of each section of the antenna to attain the UWB performance, the surface current distribution is presented in Figure 5 at different frequencies.

**Figure 6:**
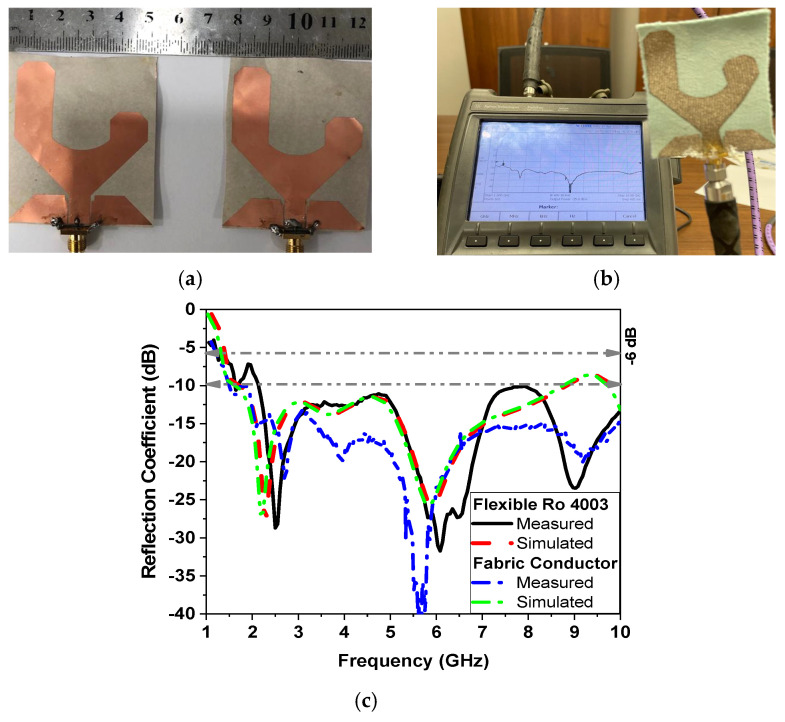
Measurement results. (**a**) Photo of the fabricated antenna. (**b**) Measurement setup. (**c**) Simulated and measured reflection coefficient.

The prototype photo and reflection coefficient |S11| in dB, measured and simulated, of the rectangular monopole antenna based on the Rogers and cotton substrates are shown in Figure 6. The antenna is measured using the Agilent vector network analyzer FieldFox N9918A 26.5 GHz, as shown in Figure 6b. The proposed antenna exhibits a UWB bandwidth from 1.5 GHz to 10 GHz at |S11| ≤ −6 dB (VSWR ≤ 3), as demonstrated in Figure 6c, and from 2.2 GHz up to 10 GHz at |S11| ≤ −6 dB (VSWR ≤ 2). The deviations between the measured and simulated results could be attributed to the use of silver epoxy for soldering the connector to the flexible substrate, which is not taken into account in the simulation model. Nevertheless, the measured response of the proposed flexible Roger antenna displays a similar behavior. Both measurements and simulations show dual resonances.

## 3. Microwave Sensors with Human Chest Phantom

This section focuses on analyzing the performance of the proposed MS antennas when in contact with human chest tissues both with and without water [38,39]. In order to evaluate and investigate the impact of the textile material conductivity to detect changes in lung water level from a normal to an edematous condition, simulation experiments were initially carried out by using a 3D electromagnetic simulator HFSS (Ansys Ver. 15) [36]. Two different types of microwave sensors are applied in this experiment. By changing the fractional lung tissue volume from 20% (normal lung) to 40% (edematous lung), variations in lung water level are modeled. A simplified multilayer phantom model of the lung and muscular tissue is simulated over the operating sensors’ frequency bands using the same two types of microwave sensors placed side by side [40].

**Figure 7:**
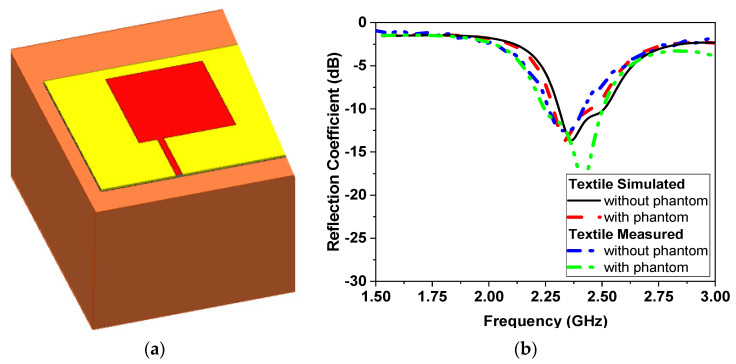
The model of a human chest with and without the phantom of the textile sensor (**a**) Chest phantom. (**b**) |S11| response.

To conduct this analysis, a simulation model is utilized, which includes a three-layer phantom model for the human chest, as well as an additional layer for water. The dimensions of the three-layer model are 25 *×* 20 *×* 12 cm^3^, as illustrated in Figure 7a, and consist of skin, fat, and muscle glandular tissues, as outlined in [40]. To reduce computation time, a one-effective-layer phantom is also implemented in the simulations, which has an effective dielectric constant of ε_r_ = 33, an effective conductivity of σ = 2 S/m, and a thickness of 120 mm, as mentioned in [41,42].

### 3.1. Narrowband Microwave Sensors

**Figure 8:**
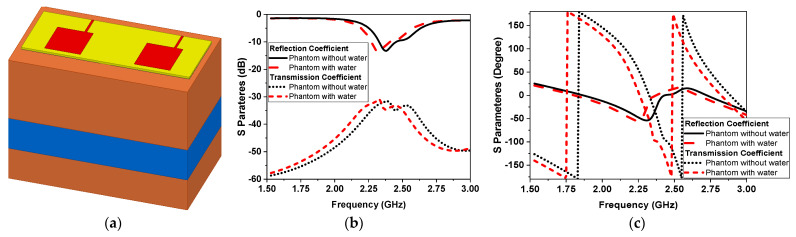
Simulation of two NB microwave sensors. (**a**) The 3D model. (**b**) Magnitude of |S11| in dB. (**c**) |S11| phase (red without water level, black with water level).

**Figure 9:**
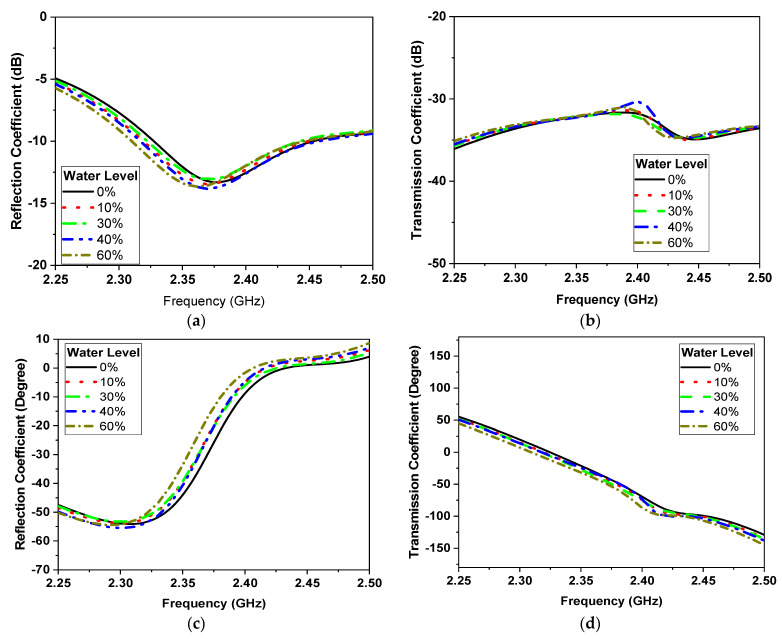
Simulation of the S parameter’s magnitude. (**a**) Reflection coefficient. (**b**) Transmission coefficient and phase. (**c**) Reflection coefficient. (**d**) Transmission coefficient of the NB sensor.

The one-effective-layer phantom reflection coefficient magnitude and phase are simulated and compared with the measured phantom, as shown in Figure 7. The reflection coefficient curves |S11| of the proposed textile antenna using a practical human chest and approximation models show reasonable agreement, as illustrated in Figure 7b. Due to the effect of the human chest, the resonance frequency is shifted down from 2.35 GHz to 2.3 GHz. We notice that the maximum reflection coefficient |S11| has a value of −14 dB at the resonance frequency of 2.3 GHz. The maximum bandwidth at |S11| ≤ −10 dB of the proposed antenna was 1.25% (30 MHz). The second step of the design to extract the human vital signs is adding another microwave sensor placed side by side, as shown in Figure 8a. Another layer is added in the simulation as water layer with a thickness of 2 cm, at 10 cm from the chest surface, and then the S-parameters of the two sensors are calculated and measured to diagnose the water level, as shown in Figure 8b,c. Figure 8a shows the proposed experiment to monitor the water level in the human lung using two sensors, while Figure 8b shows the difference in reflection and transmission coefficient with and without water in the human lung; the S-parameter magnitude shows that the resonant frequency shifted down by 20 MHz and 0.5 dB in the magnitude while the phase difference is about $50^{o}$. Figure 9 shows the variation of the water level in the human lung from 0% to 60% on the sensor parameters, with a frequency shift from 2.3 GHz to 2.275 GHz, a magnitude shift of the S parameters by 1.5 dB, and a phase shift by 5 degrees.

### 3.2. Ultra-Wideband Microwave Sensors

**Figure 10:**
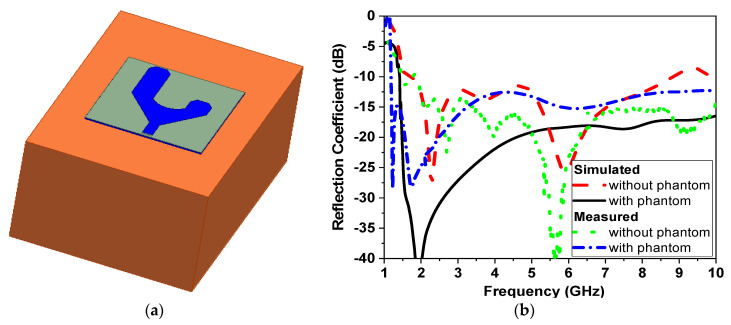
UWB microwave sensor. (**a**) Phantom model. (**b**) Reflection coefficient magnitude.

**Figure 11:**
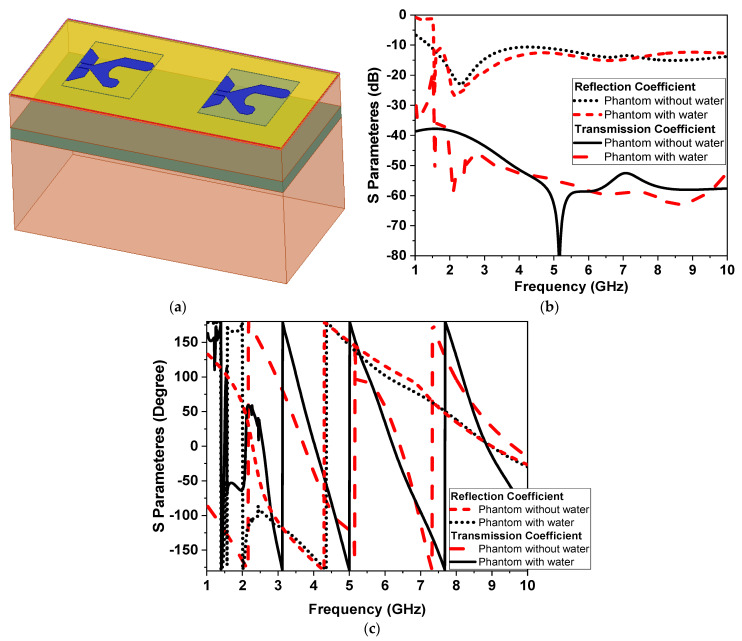
Simulation of two monopole antennas with a chest model. (**a**) The 3D model. (**b**) The S parameters’ magnitude in dB. (**c**) The S-parameters’ phase in degrees.

The |S| parameter curve as a reflection coefficient of the proposed microwave sensor on the human chest is examined by using a one-effective-layer phantom as shown in Figure 10. This figure shows that there is a good agreement between measured and simulated results. as Also, when the antenna is applied on the human chest, the resonant frequency is shifted down and the magnitude of the reflection coefficient is changed due to the effects of the human load on the microwave sensor. As in the previous scenario of the design to extract the human vital signs, we added another microwave sensor placed side by side, as shown in Figure 11a. Another layer is added in the simulation as water layer, with a thickness of 2 cm, at 10 cm from the chest surface, and then the S-parameters of the two sensors are calculated and measured to diagnose the water level, as shown in Figure 11b,c.

**Figure 12:**
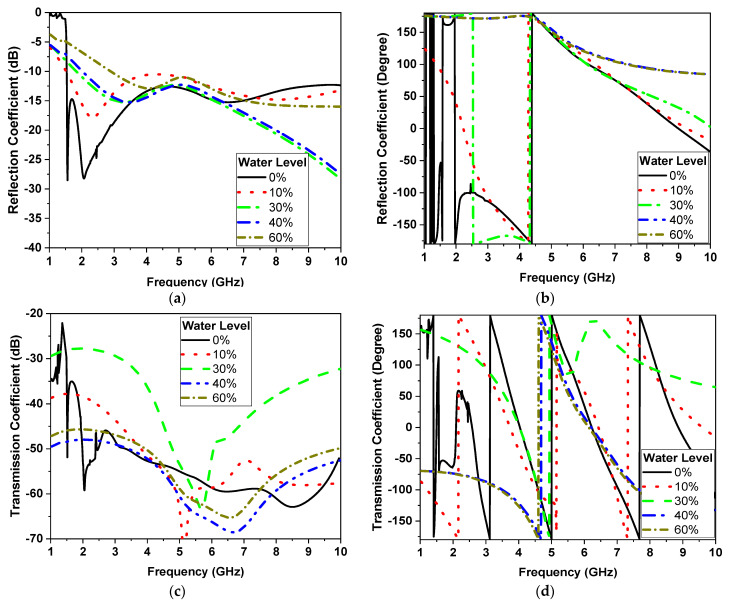
Simulation of the S parameters’ reflection coefficient (**a**) magnitude and (**b**) phase, and transmission coefficient (**c**) magnitude and (**d**) phase.

Figure 12 shows the results of the water level percentage of the UWB sensor. These results show the huge difference over the operating band on the S parameters’ magnitude and phase. As the water level in the human lung increased, the reflection and transmission coefficient magnitude reduced on average over the operating band by 5 dB and 10 dB, respectively, while the reflection and transmission coefficient phase reduced by 25° and 50° on average, respectively.

## 4. Specific Absorption Rate Measurement

The SAR is defined as the amount of power absorbed per unit mass of tissue, as reported in [43]. This falls below the in-safety limits for calculated SAR over 1 g and 10 g of tissue of 1.6 W/kg and 2 W/kg, respectively, according to the IEEE C95.3 standard [43]. The SAR is measured by placing the two different types of textile antenna microwave sensors (NB and UWB) on the phantom tissue model R&S CMX500 OBT (SPEAG DASY8) system for SAR. The SAR was measured at a frequency of 2.45 GHz, as shown in Table 3, for the value of 1 g and 10 g at different transmitted powers of 10, 15, and 20 dBm, respectively. The value of the SAR of the NB sensor is less than that of the ultra-wide bandwidth at the head and body. Moreover, both types of sensors achieved the safety SAR levels for the proposed textile sensor as long as the transmitted power did not exceed 100 mW. Thus, the proposed two types of microwave sensors could be embedded in wearable applications.

## 5. Proposed System for Vital Sign Detection

In this section, we present a wearable electronic system that can track vital signs like respiration rate and heart rate. The sensors are flexible to bend to the patient chest, and the mechanical movement of the chest generates variation in the transmission coefficient phase when the patient breathes. To extract the respiratory pattern, the system investigates the transmission coefficient phase at a specific frequency versus time.

**Figure 13:**
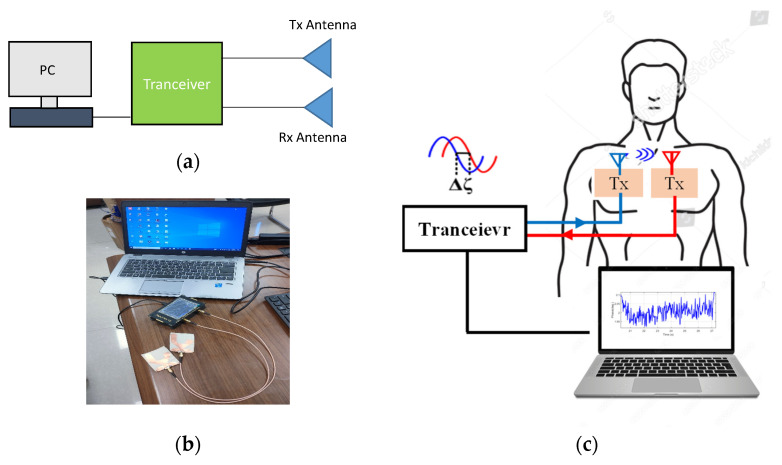
The proposed system. (**a**) Block diagram. (**b**) System photo. (**c**) Experiment setup.

The sensor’s main feature is that it converts breathing movements into phase variations in RF wave impulses, making it extremely resistant to external interference. The proposed system block diagram is shown in Figure 13.

The vital sign detection block diagram represents a system for detecting vital signs using two sensors, a Tx and an Rx, a nano VNA acting as a transceiver, and a laptop for data acquisition and analysis. The Tx sensor transmits a signal towards the body, and the Rx sensor receives the signal passed through the body. The received signal is analyzed to extract information about vital signs. The nano VNA measures the signals transmitted and received by the sensors, while the laptop controls the process and acquires the data. This basic system can be customized to fit specific applications.

### 5.1. Principle of Operation

**Figure 14:**
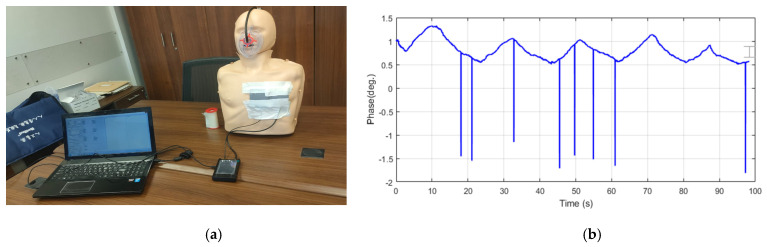
Proof of concept. (**a**) Measurement setup. (**b**) Breathing behavior versus time.

Assessing the respiratory rate can be efficiently done by observing the mechanical displacement that occurs on the thoracic wall resulting from breathing. To identify this repetitive motion, we utilize the properties of electromagnetic waves as they travel through the air to determine the breathing rate. Additionally, we analyze the way these waves propagate through the body to identify the rate of the heartbeat. The approach we employ involves measuring the displacement by making phase variation measurements on sensors. In this study, we suggest bending the sensors towards the patient’s chest to take advantage of the phase variation created by the distance between them. During inhalation, the chest expands, and the sensors move dynamically in response. During a regular daily silent breathing state, the chest expansion movement is estimated to be between 0.1 mm and several millimeters [5]. The thoracic wall glides back and forth during a calm heartbeat. Between the transmitting Tx and the receiving Rx sensor, we continually transmit a sine wave at 2.45 GHz and compare the phases at both ends. The lungs make a repeated movement by breathing in and out, causing the initial distance between the two sensors to fluctuate. We can derive a breathing pattern by observing the phase variation with each displacement. Initially, the concept described above is tested using an artificial human body, as illustrated in Figure 14. An artificial human body is chosen to replicate the shape and size of a human chest. This artificial body is made from a plastic material that allows the transmission of electromagnetic waves. Two identical sensors are positioned on the artificial body’s chest, aligning them symmetrically with the chest area. The sensors are connected to a receiver or signal processing system capable of detecting and analyzing electromagnetic waves. A pump and an air bag, designed to mimic the shape of a human chest, are utilized. The air bag is placed inside the artificial body and connected to the pump. By inflating and deflating the air bag, the pump simulates the motion of breathing. The experiment begins by activating the pump to initiate the inflation and deflation of the air bag. As the air bag expands and contracts 5 times/100 s, it mimics the movement of the chest during breathing. Simultaneously, the sensors capture the electromagnetic waves transmitted through the artificial body. The received signals from the sensors are analyzed to extract the breathing rate. The changes in the received signal caused by the chest movement during breathing are reflected in the data collected by the sensors. By examining variations in signal phase, it becomes possible to estimate the breathing rate. The breathing rate is presented in Figure 14b. It is observed that the breathing pattern correlates well with the actual chest motion.

### 5.2. Sensor Assessment in Vital Sign Detection

In this section, we are going to evaluate the performance of both the NB and the UWB sensor in the application of monitoring vital signs, namely, breathing rate, heartbeat rate, and lung water level.

#### 5.2.1. Breathing Rate

In the upcoming sections, we will evaluate the suggested sensors in terms of their ability to detect breathing rates.

##### Narrowband Sensor

The NB sensor is used as a Tx and an Rx sensor. The distance between the Tx sensor and the Rx sensor is examined. Then, several experiments are conducted for two different cases, an adult male of 35 years old, and a female child of 8 years old. The data gathered give enough sensitivity and accuracy to state that the device can work with different physical morphologies. In the following subsections, the experiment results will be detailed.

**Figure 15:**
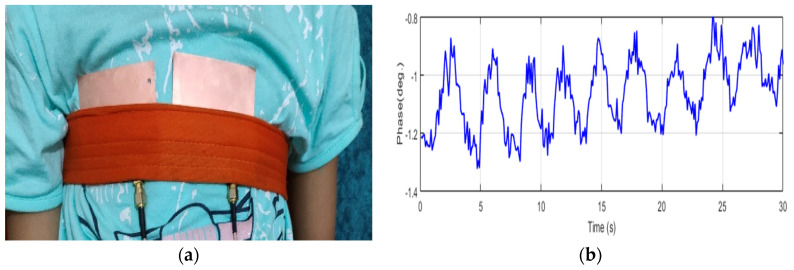
Breathing rate for the 8-year-old child using NB sensors. (**a**) Experiment setup. (**b**) Phase versus time.

**Figure 16:**
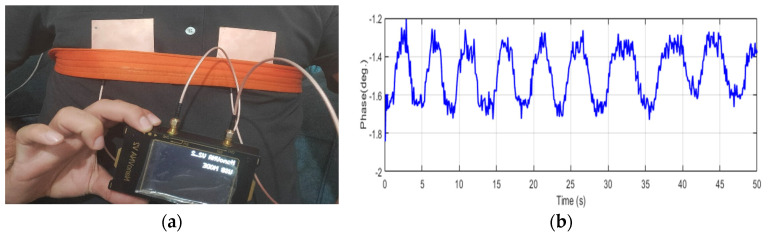
Breathing rate for the 35-year-old adult using NB sensors. (**a**) Experiment setup. (**b**) Phase versus time.

**Figure 17:**
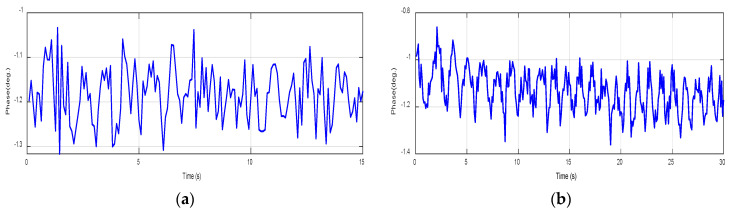
Breathing rate after a running activity using NB sensors. (**a**) Child case. (**b**) Adult case.

Figure 15 and Figure 16 demonstrate the normal breathing rate. For the child, it shows around 20 breaths/min while in the adult case, it is reduced to 12 breaths/min, which is perfect. To test the system sensitivity, we repeated the experiment after a running exercise. The breathing behavior is demonstrated in Figure 17a,b for both the child and the adult. It is clear that the breathing rate increased to 60 breaths/min and 48 breaths/min for the child and the adult, respectively.

##### UWB Sensor

To evaluate the performance of the UWB sensor, we repeated the previous experiments after connecting the proposed UWB sensor to the system. The same adult and child who were subjected to the first experiments were asked to help in the current experiment.

**Figure 18:**
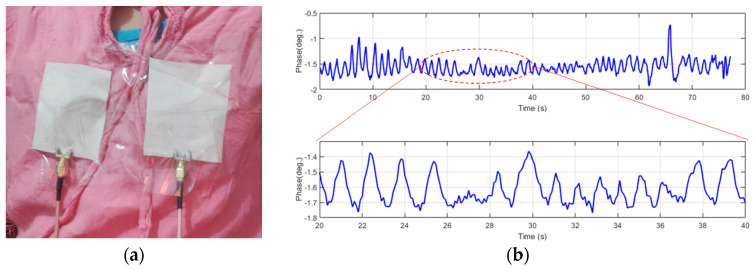
Breathing rate for the 8-year-old child in normal conditions using UWB sensors. (**a**) Experiment setup. (**b**) Phase versus time.

**Figure 19:**
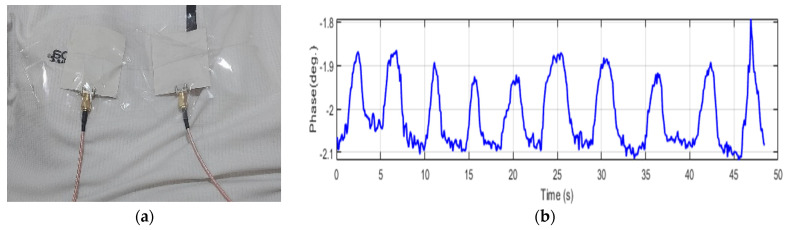
Breathing rate for the 35-year-old adult in normal conditions using UWB sensors. (**a**) Experiment setup. (**b**) Phase versus time.

The experiment setup and breathing rate for the two instances, child and adult, are shown in Figure 18 and Figure 19. To extract the normal breathing rate, the two persons were asked to sit down and breathe normally. The child’s breathing rate was around 21 breaths per minute, while the adult’s was around 12 breaths per minute, indicating that the child’s breathing rate was higher than the adult’s. The proposed device performs perfectly when compared to the typical breathing rate.

**Figure 20:**
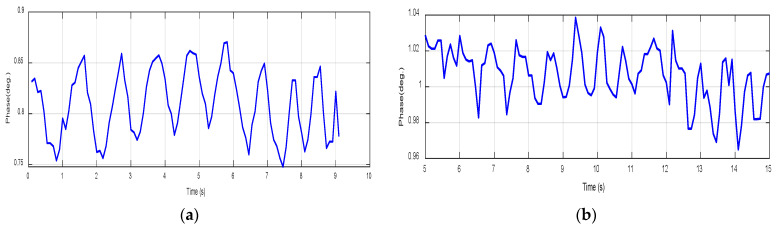
Breathing rate after a running activity using UWB sensors. (**a**) Child case. (**b**) Adult case.

In the second experimental phase, prior to commencing data acquisition, we instructed both participants to engage in a jogging exercise to evaluate the sensitivity of the proposed device. Figure 20 illustrates the results for both the child and the adult, revealing elevated breathing rates in both scenarios: 78 breaths per minute for the child and 60 breaths per minute for the adult. This suggests that the sensor exhibits the capability to detect respiratory rates exceedingly fast.

#### 5.2.2. Heartbeat Rate

**Figure 21:**
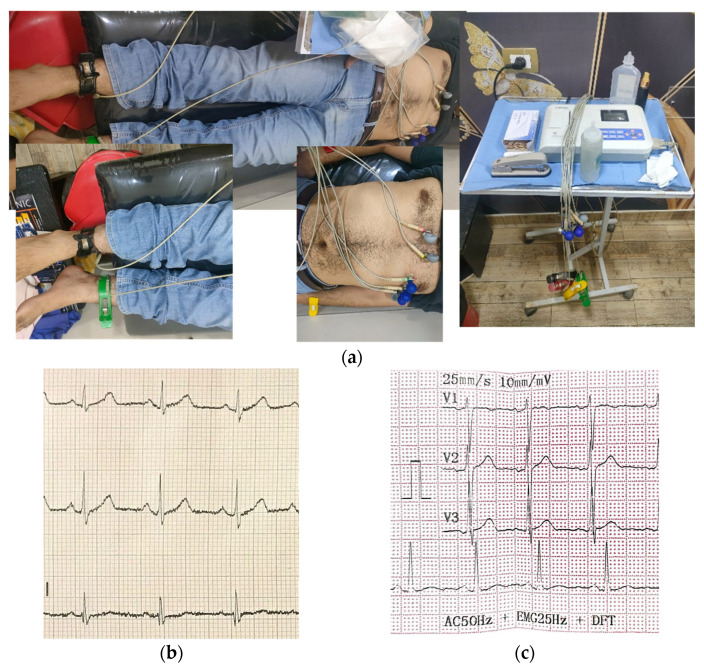
Screenshot of ECG measurement using the CONTEC Machine Electrocardiograph ECG100G. (**a**) Measurement setup. (**b**) Child ECG report. (**c**) Adult ECG report.

In this section, we describe the evaluation of sensors for heartbeat rate extraction, covering both narrowband and UWB sensors. The measurement of the heartbeat rate using a commercial device (the CONTEC Machine Electrocardiograph ECG100G) serves as a reference for our proposed device performance assessment. In this context, the ECG heart rate measurements were performed on the same two volunteers, a child and an adult, utilizing the commercial device for validating the accuracy and functionality of our proposed device. Figure 21a depicts the measurement configuration, where electrodes are commonly placed on the volunteer’s skin to record electrical impulses from different locations on the body. We will employ this well-established commercial device as a reference point to evaluate and compare the performance of both narrowband and UWB sensors, as we will discuss in the subsequent sections. The recorded heart rates were 87 beats per minute (bpm) for the child and 90 bpm for the adult, as shown in Figure 21b,c.

##### Narrowband Sensor

**Figure 22:**
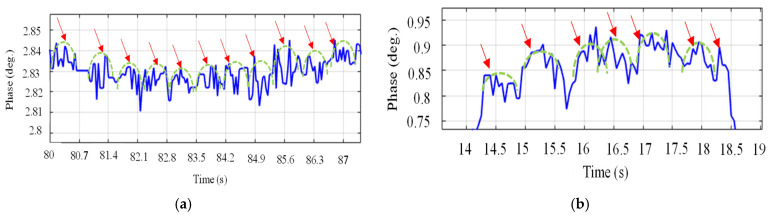
Heartbeat using NB sensors. (**a**) Child case. (**b**) Adult case.

To assess the ability of the narrowband sensor ability to detect heart rate, the same volunteers were instructed to briefly suspend their breath while maintaining fully inflated lungs. The purpose of this instruction was to eliminate any phase variations induced by breathing, thereby isolating the heartbeat signal. As shown in Figure 22a, in the case of a child, we observe a phase with repetitive oscillations, indicating pulse movements, and no simultaneous thoracic motion related to respiration. Nevertheless, the narrowband sensor’s performance approximately yielded a heartbeat rate estimate of 85.7 bpm for the child. In Figure 22b, we present the heartbeat of the adult volunteer, demonstrating a heartbeat rate of 93 bpm. It is worth noting that the influence of the heartbeat on phase changes is less pronounced in this case. The child’s heart rate induces a phase change of approximately 0.01 degrees, whereas in the case of the adult, it fluctuates about 0.05 degrees. Furthermore, when comparing these results to those obtained with the commercial device, a deviation of 1.5 bpm for the child and 2 bpm for the adult is observed.

##### UWB Sensor

**Figure 23:**
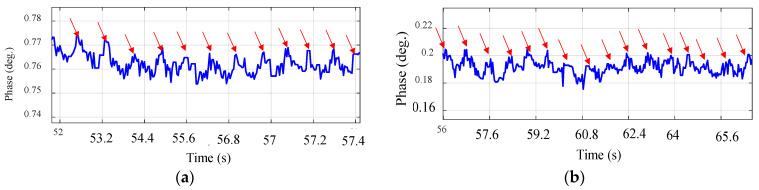
Heartbeat using UWB sensors. (**a**) Child case. (**b**) Adult case.

The respiratory patterns for both scenarios, involving a child and an adult, are presented in Figure 23. Notably, when holding one’s breath with fully inflated lungs, the heart rate oscillations become distinctly prominent. The conducted experiments provided profoundly convincing evidence regarding the effectiveness and viability of the UWB sensor. In the case of the child, the heart rate registers at 86 bpm, as shown in Figure 23a, while in the adult case, it is 92.3 bpm, as shown in Figure 23b, almost aligning with commercial device results for each case. It is noticed from Figure 23 that the child’s heart rate leads to a phase shift of roughly 0.015 degrees, while for the adult the phase shift is 0.02 degrees. Moreover, when comparing these findings with the measurements from the commercial device, a slight discrepancy of 1 bpm for the child and 1.3 bpm for the adult becomes apparent. To summarize the results, Table 4 illustrates a comprehensive comparative analysis between the UWB textile sensor, the NB textile sensor, and the commercialized system (CONTEC Machine Electrocardiograph ECG100G). Remarkably, the data reveal a notable proximity between the UWB textile sensor in our proposed design and the commercialized system, surpassing the performance of the narrowband textile sensor. This finding underscores the promising potential of UWB technology in textile-based vital sign detection systems, approaching the standards set by established commercial solutions.

## 6. Lung Water Level Monitoring

Monitoring lung water levels is crucial for the early detection and management of various medical conditions, such as congestive heart failure and pulmonary edema. Traditional methods for assessing lung water levels often involve invasive procedures, which can be uncomfortable and risky for patients. Non-invasive techniques, such as using microwave-based sensors with narrowband and UWB sensors, offer a promising alternative. This section explores the application of narrowband and UWB sensors for measuring LWL and compares their performance based on frequency shift analysis.

### 6.1. Lung Phantom Preparation: Mimicking Human Lung Tissue

**Figure 24:**
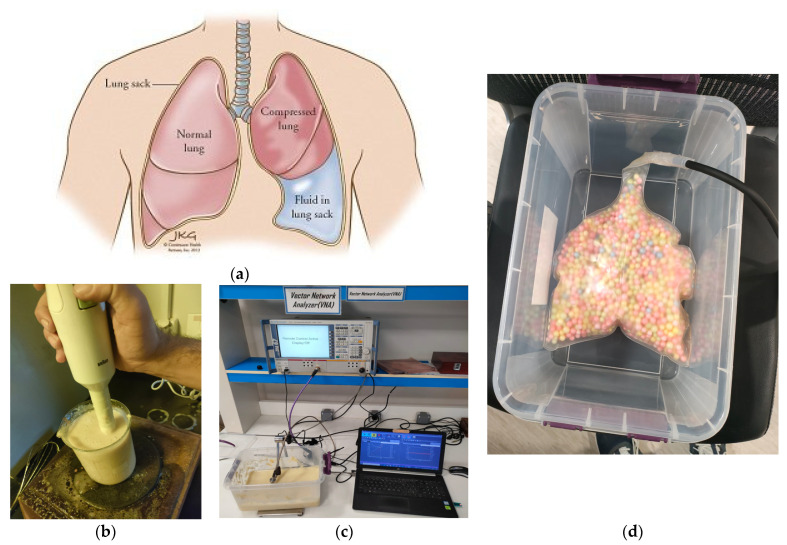
(**a**) Drawing showing the water in the human lung [44]. (**b**) Phantom preparation and fabrication. (**c)** Measurement. (**d**) Photo of the artificial plastic lung filled with foam.

Creating an effective lung phantom that closely mimics the permittivity of human lung tissue, as shown in Figure 24a, is of paramount importance for the precise evaluation of our sensor technology. Figure 24b shows the phantom preparation process. Our meticulous preparation process involves several steps to achieve this goal. Initially, in a substantial beaker, we combine 1.5% agar powder with 500 mL of distilled water and heat the mixture until it reaches boiling point, ensuring the complete dissolution of the agar through continuous stirring. Concurrently, in another beaker, we mix 5% gelatin powder with 250 mL of distilled water, subjecting it to heat and rigorous stirring until the gelatin achieves full dissolution.

To enhance the lung phantom’s likeness to human lung tissue, we introduce 5 g of sodium chloride into the agar mixture, ensuring its thorough dissolution. Additionally, 5 g of sodium azide is incorporated into the gelatin mixture, effectively dissolving it. Subsequently, we merge the gelatin mixture into the agar mixture, ensuring comprehensive blending. To further refine the phantom, we add 150 mL of propylene glycol and 50 mL of safflower oil, followed by thorough stirring. Achieving the desired consistency involves the meticulous integration of 50 g of corn flour and 2 g of xanthan gum into the solution. To enhance realism, we incorporate 50 g of polyethylene powder, thoroughly mixing it into the mixture.

Ensuring optimal results and homogeneity, we introduce a small amount (less than 1 mL) of liquid detergent, gently incorporating it. Maintaining a pH level of 6.8 is crucial to achieve the desired characteristics, with adjustments made using NaOH or HCl solutions. To fine-tune permittivity precisely, we introduce deionized water, adjusting the water content as necessary. Further refinement occurs through heating the mixture to 50–60 °C, effectively dissolving any residual salt crystals while promoting thorough mixing. The resulting blend is then introduced into a mold, left to cool, and afterward solidify. It is then assessed using a DAK 3.5 (200 MHz–20 GHz) device at the ERI laboratory in Egypt with high measurement repeatability (typ. within ±1%) accuracy. Lastly, we introduce this blend into artificial plastic lung-shaped structure filled with foam that is used to replicate the air within the lung, as depicted in Figure 24b. In our frequency range band, the relative permittivity of the lung mimic values lies between 32 and 45, while the effective conductivity of the lung falls within the range of 0.9 to 2.2 S/m. These findings are in close alignment with the relative permittivity range of 20 to 53 and effective conductivity range of 0.3 to 2.1 S/m for the human chest as documented in [45].

#### 6.1.1. Narrowband Sensor

Narrowband sensors are commonly used in medical applications due to their simplicity and ease of implementation. These sensors emit electromagnetic waves at a specific frequency, which propagate through the body. When these waves encounter different dielectric properties, such as the transition from air to lung tissue or water, they experience a frequency shift. This shift is caused by the varying propagation speed of electromagnetic waves in different materials. The most straightforward way to measure the water level in the lung is the frequency and amplitude shift analysis which involves transmitting a known frequency signal through the human chest. When the signal crosses the lung tissue–water interface, it experiences a significant frequency/amplitude shift due to the difference in dielectric properties. This shift is directly related to the amount of water present in the lungs.

**Figure 25:**
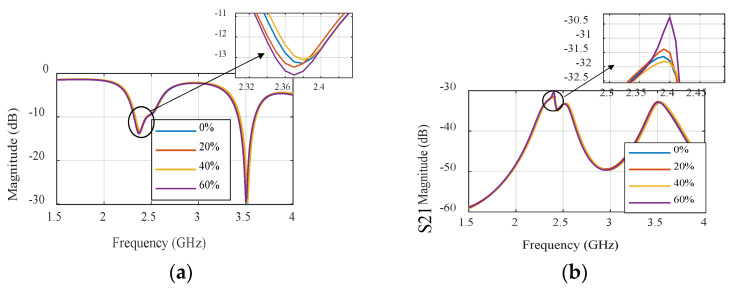

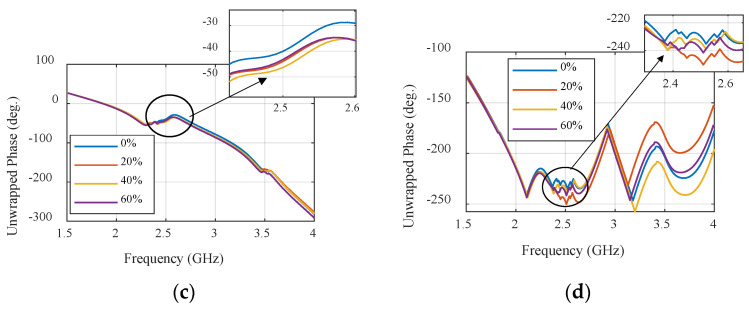
Simulated S-parameters of different water contents for the narrowband sensor: (**a**) S11 magnitude. (**b**) S21 magnitude, (**c**) S11 unwrapped phase, and (**d**) S21 unwrapped phase.

**Figure 26:**
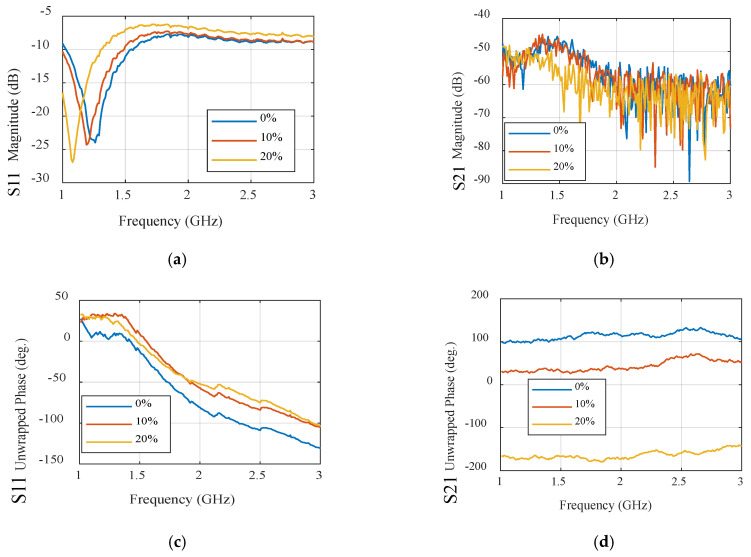
Measured S-parameters of different water contents for the narrowband sensor. (**a**) S11 magnitude. (**b**) S21 magnitude. (**c**) S11 unwrapped phase. (**d**) S21 unwrapped phase.

Figure 25 and Figure 26 serve to present a comprehensive analysis of the simulated and measured S-parameter characteristics as a function of frequency, conducted with two narrowband sensors working as Tx/Rx and subjected to varying water levels. Specifically, Figure 25a showcases the sensor’s decreasing ability to detect alterations in both the frequency and amplitude components of the reflection coefficient, across a range of water content percentages from 0% to 60%. Correspondingly, Figure 25b highlights the sensor’s capacity to observe significant shifts in the frequency and amplitude of the transmission coefficient, denoted as S21. Moreover, Figure 25c,d, respectively, illustrate the phase responses of the reflection coefficient, S11, and the transmission coefficient, S21, with reference to the same range of water levels.

It is pertinent to note that the designed narrowband sensor demonstrates its proficiency in quantifying water levels through discernible alterations in either the amplitude or phase characteristics of S11 and S21. Notably, the phase shift exhibits heightened sensitivity compared to amplitude variations, further underscoring the sensor’s efficacy for precise water level measurement. The measurements were carried out employing the phantom prepared in the preceding subsection, and the same observations are evident in the measurement curves depicted in Figure 26. It is apparent that the measured results in the experimental model are highly affected by increasing the water level due to ignoring some factors in the simulation process such as human bones, tissues, etc.

#### 6.1.2. UWB Sensor

**Figure 27:**
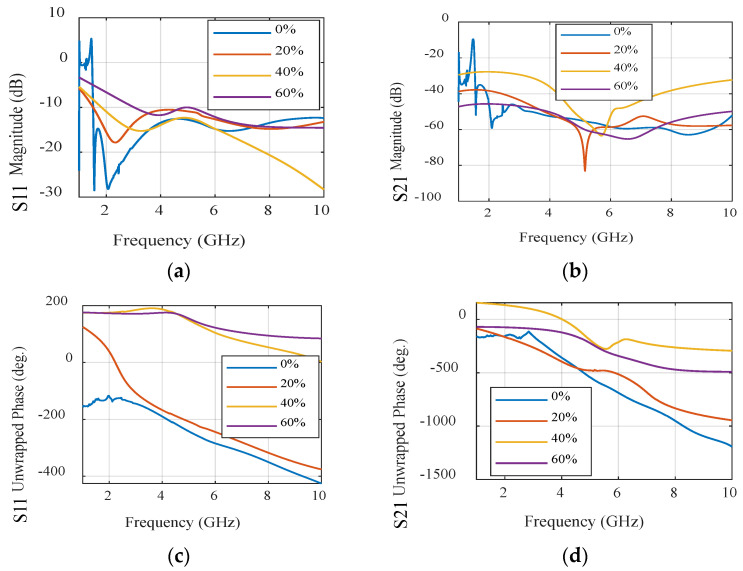
Simulated S-parameters of different water contents for the UWB sensor. (**a**) S11 magnitude. (**b**) S21 magnitude. (**c**) S11 unwrapped phase. (**d**) S21 unwrapped phase.

**Figure 28:**
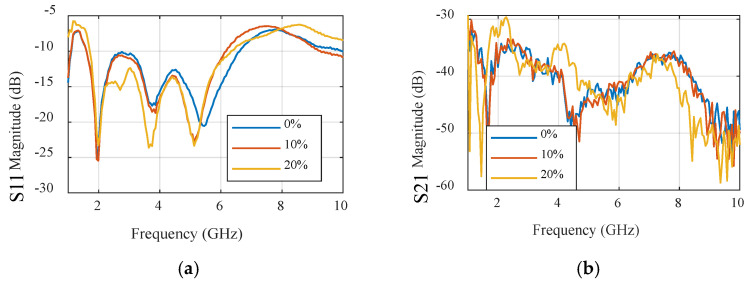

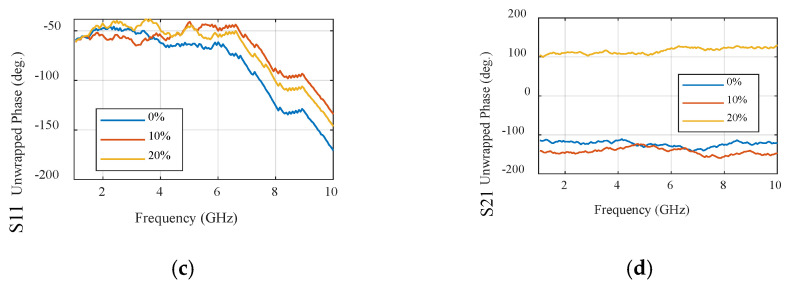
Measured S-parameters of different water contents for the UWB sensor. (**a**) S11 magnitude. (**b**) S21 magnitude. (**c**) S11 unwrapped phase. (**d**) S21 unwrapped phase.

In contrast, when applying the same set of experiments to the UWB sensor, we can draw parallel conclusions. The results of these experiments are showcased in Figure 27 and Figure 28, encompassing sub-figures labeled as a, b, c, and d, similar to Figure 25 and Figure 26. These figures elucidate how the UWB sensor excels in measuring water levels using S-parameter characteristics as functions of frequency. Notably, the UWB sensor demonstrates a superior level of detectability compared to the narrowband sensor, allowing it to detect even finer level of water levels. Additionally, the phase responses of the reflection coefficient (S11) and transmission coefficient (S21) across various water content percentages reveal the UWB sensor’s measurements’ remarkable precision.

This comparison not only highlights the applicability of both sensor types to water level measurement but also underscores the distinct advantages of the UWB sensor, particularly in terms of sensitivity and precision.

## 7. Discussion and Comparisons

According to the previous results of the vital signs and LWL measurements, we can conclude that the UWB antennas offer several advantages over narrowband sensors. The ultra-wide band antennas offer a stable phase over frequency causing a very high accuracy in breathing and heartbeat rate monitoring. Furthermore, UWB signals can penetrate tissues more effectively and provide a clearer distinction between different materials within the body. Consequently, UWB sensors can offer a more precise measurement of LWL.

The comparative study between NB and UWB textile antenna sensors for vital sign detection and lung water measurement reveals promising applications in healthcare and the daily lives of athletes. The superior performance of UWB technology suggests seamless integration into healthcare systems, enabling accurate and reliable continuous monitoring. UWB sensors, being non-invasive, enhance patient comfort, promote compliance, and facilitate long-term monitoring for chronic conditions. The technology allows for remote patient monitoring, empowering healthcare providers to track and manage patient health remotely. Moreover, these wearable devices could be adapted for home use, enabling individuals to monitor vital signs and lung water levels at home, promoting a proactive approach to health management and potentially reducing the need for frequent clinic visits.

Table 5 presents a detailed comparative analysis between NB and UWB textile sensors, highlighting key features, trade-offs, and considerations to deepen the understanding of their respective strengths and applications. Furthermore, Table 6 presents a comparison of different studies [26,27,28,29,30] and a new proposed work in the context of NB wearable sensor designs. The key parameters compared include sensor material, sensing mechanism, frequency, size, SAR value, and data post-processing technique. It is clear that the proposed design has a smaller size than [26,30], and has the smallest SAR value except [27]. Table 7 provides a comparison between the proposed wearable breathing sensor and some other reported sensors. The key parameters compared include sensor material, sensing mechanisms, bandwidth, size, sensitivity, and data postprocessing technique. The proposed sensor is fabricated using a combination of PCB (printed circuit board) and textile materials. Also, it obtains a smaller size and a wider bandwidth. It is worth noting that the antennas used in previous studies necessitated the integration of stretchable or compressible elements to detect breathing rates, resulting in a substantial gain change (measured in dB).

## 8. Conclusions

This study explored the field of wearable MAS for the purpose of vital sign detection (VSD) and LWL monitoring. The investigation scrutinized two distinct categories of MAS, NB and UWB, with the aim of discerning their comparative efficacy. These wearable antennas distinguish themselves by their simplicity, low-profile design, and cost-effectiveness, making them promising candidates for the development of wearable health-monitoring devices. Crucially, the study adhered to rigorous safety standards, measuring the SAR of the sensors at various resonant frequencies under both 1 g and 10 g tissue conditions, in accordance with the IEEE C95.3 standard. Remarkably, both sensor types demonstrated compliance with the standard limits of 1.6 W/kg and 2 W/kg, respectively, ensuring their safety for use in proximity to the human body.

This investigation not only underscores the promising potential of NB and UWB MAS for wearable health monitoring but also highlights the UWB sensor’s superior efficiency in achieving highly sensitive and accurate measurements. These findings provide a strong foundation for the development of innovative and cost-effective wearable healthcare devices, with the UWB technology standing out as a particularly compelling choice for enhancing patient care and well-being.

## Figures and Tables

**Table 1:** The optimized dimensions of the proposed NB antenna (all dimensions in mm).

Rogers Flexible Substrate						
L_S_	W_f_	L_p_	Ws	L_f_	W_p_	X_f_
50	30	35	50	12	32	1.5
Textile substrate						
60	24	35	60	20	32	6

**Table 2:** The optimized dimensions of the proposed UWB antenna (all dimensions in mm).

*L_s_*	*L_f_*	*L_m_*	*L_k_*	*L_p_*	*L_gg_*	*W*_*p*1_	*S*
60	10	26	10	22	2	19.6	0.5
*W_s_*	*W_f_*	*W_m_*	*Lg*	*W_p_*	*W_gg_*	*W_m_*_1_	*-*
60	6	12.7	10	44	2	6.7	-

**Table 3:** The measured SAR of narrowband and UWB microwave sensors.

Narrowband Microwave Sensor				Ultra-Wideband Microwave Sensor		
2.45 GHz		SAR (1 g)	SAR (10 g)	2.45 GHz	SAR (1 g)	SAR (10 g)
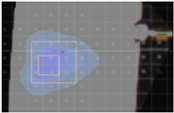		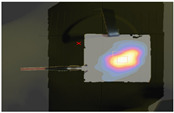		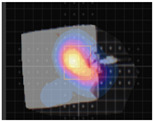	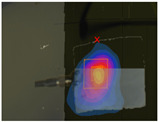	
Head	10 dBm	0.088	0.044	10 dBm	0.25	0.124
	15 dBm	0.274	0.138	15 dBm	0.794	0.393
	20 dBm	0.887	0.447	20 dBm	1.83	1.25
Body	10 dBm	0.093	0.042	10 dBm	0.329	0.137
	15 dBm	0.304	0.137	15 dBm	1.04	0.435
	20 dBm	1.02	0.448	20 dBm	1.91	1.39

**Table 4:** Comparative performance analysis of the complete system and proposed design for vital sign detection.

Heartbeat Rate	CONTEC Machine Electrocardiograph ECG100G	Proposed System Using NB Sensors	Proposed System Using UWB Sensors
37-year-old adult	90 bpm	93 bpm	92.3 bpm
8-year-old child	87 bpm	85.7 bpm	86 bpm

**Table 5:** Comparative analysis of NB and UWB textile sensors.

Feature	NB Sensor	UWB Sensor
Power Consumption	Higher	Lower
Data Transmission Rates	Slower	Faster
Real-World Usability	Limited Range	Expanded Range
Cost	Lower	Higher
Applications	Specific	Diverse
Signal Processing	Specific	Diverse
Accuracy	Moderate	High

**Table 6:** Comparison of the proposed NB antenna with previously published work.

Ref	Antenna Material	Sensing Mechanism	Frequency (GHz)	Size (mm^2^)	Max. SAR (W/kg)	Data Post-Processing
[26]	Textile (Denim)	Dielectric sensing	2.45	35.4 × 82.4	0.295	Peak detection
[27]	ShieldIt Super/Felt	Dielectric sensing	2.4	80 × 20	0.0091	N/A
[29]	Textile	Dielectric sensing	2.45	40 × 20	NA	N/A
[30]	Leather and Textile	Dielectric sensing	2.45	120 × 120	0.04	N/A
This Work	PCB and Textile	Dielectric sensing	2.3	50 × 50	0.042	Peak detection

**Table 7:** Comparison of the proposed UWB antenna with previously published work.

Ref	Antenna Material	Sensing Mechanism	Bandwidth (GHz)	Size (mm^2^)	Sensitivity	Data Post-Processing
[16]	Textile	Dielectric sensing	0.5-3	118 × 105	1.5–2.5 dB *	Peak detection
[21]	PCB	Dielectric sensing	N/A	N/A	10% *	N/A
[2]	Stretchable Textile	Strain sensing	0.4–1.4	120 × 30	1.5–7 dB &*	N/A
This Work	PCB and Textile	Dielectric sensing	2–10	60 × 60	0.017 **	Peak detection

* Amplitude sensitivity, ** Phase sensitivity.

## Data Availability

Data are contained within the article.
